# Nutritional outcomes in edentulous patients with conventional and single-implant overdentures: A cross-sectional study

**DOI:** 10.6026/973206300220224

**Published:** 2026-01-31

**Authors:** Vishal B Parmar, Birood G Patel, Brinda Patel, Diptesh S Rami, Ravindra M Chavda

**Affiliations:** 1 Department of Prosthodontics and Crown & Bridge, AMC Dental College & Hospital, Ahmedabad, Gujarat, India; 2 Department of Prosthodontics and Crown & Bridge, Narsinhbhai Patel Dental College and Hospital, Visnagar, Ahmedabad, Gujarat, India; 3 Private Practitioner, Shreeji Dental and Skin Care, Surat, Gujarat, India; 4 Department of Prosthodontics and Crown & Bridge, AMC Dental College & Hospital, Ahmedabad, Gujarat, India

**Keywords:** Edentulism, implant overdenture, nutritional status, pre-albumin, complete denture, elderly nutrition

## Abstract

The elderly patients who have their edentulas compromised by aging usually have impaired nutritional status because of their inability to masticate their food and have a diverse diet using standard complete dentures. The on-going prospective cross-over trial has put five fully edentulous patients (mean age 60 years) to assess and compare whether conventional complete dentures and single implant-retained overdentures have differences in their effects on sensitive nutritional markers. Nutritional assessment was done through serum pre-albumin, albumin, vitamin B12, iron and haemoglobin at the baseline (edentulous), one month of CD placement and one month of IOD placement. Compared to traditional dentures, central single implant-retained overdentures have shown to provide better nutritional result, especially in highly sensitive indices like pre-albumin and albumin indicating a better station of dietary consumption and protein-energy condition in edentulous elderly patients.

## Background:

Full edentulism has always been a widespread chronic disease in geriatric populations of the global community, which is the ultimate result of various oral diseases and overall health loss [1]. Although the prevalence is becoming less common in the developed countries, the absolute number of edentulous persons keeps rising with the process of demographic ageing and edentulism remains a major public health issue [2]. Effects of total loss of teeth are not only related to oral functioning but they have far reaching impacts on nutritional intakes, system health, psychological health and general quality of life [3]. Traditional complete dentures are the old form of treatment of edentulous patients, which are more than a century old. But, masticatory efficiency is often impaired by mandibular denture instability and the chewing performance is down to about 20-30% the natural dentition capacity [4]. This impairment has a considerable effect on the food choice and edentulous people have a preference towards softer and processed food and avoiding fibrous vegetables, fresh fruits and whole grains [5]. This has resulted in many reports of poor nutritional conditions in denture users, which is manifested by low consumption of key micronutrients such as vitamins A, C, B-complex, calcium and dietary fibre [6]. These implants transformed the nature of the prosthodontic rehabilitation as now two implant mandibular overdentures are the minimum standard of care advised by the McGill Consensus Statement on the treatment of edentulous patients [7]. Later studies have shown that the use of implant-retained overdentures has a better masticatory performance, patient satisfaction and oral health-related quality of life than conventional dentures [8]. New studies have started investigations related to the nutritional outcomes after implant therapy and randomized controlled trials have shown some positive results related to the quality of the diet and certain biochemical markers [9, 10]. Nonetheless, the necessity to install several implants is an overwhelming challenges to most patients especially the developing economies and the poor. This financial limitation has been generating the desire to explore minimalist- implantation procedures, namely the central single implant-retained overdenture concept. Single midline implants have shown positive results in preliminary clinical trials showing a survival rate of over 95% after a five-year follow-up [11, 12]. The mandibular symphysis is a perfect anatomy location because of thick cortical bone, sufficient size and proximity to vital organs, which allows predictable osseointegration using early loading protocols [13]. There is no literature on thorough nutritional evaluation after single implant overdenture despite positive clinical outcomes. Past nutritional research has mostly been done on two-implant regimens or has based their research on dietary questionnaires only with no biochemical confirmation [14]. Nutritional biomarkers derived in blood represent objective, quantifiable measurement, which is not subject to patient recall bias and cognitive impairment. Namely, serum pre-albumin can be used as a sensitive measure of recent nutritional changes because it has a short half-life of about 48 hours, whereas albumin, iron, vitamin B12 and haemoglobin can be considered as complementary measures of the whole nutritional status and deficiencies in particular [15]. Therefore, it is of interest to compare and evaluate changes in nutritional status before and after rehabilitation in totally edentulous patients with conventional complete dentures or central single implant-retained mandibular overdentures using objective blood biochemical markers.

## Materials and Methods:

## Study design and ethical approval:

This prospective cross-over clinical study was conducted at the Department of Prosthodontics and Crown & Bridge, K.M. Shah Dental College & Hospital, Vadodara, between 2013 and 2015. The study protocol received approval from the Institutional Ethics Committee, Sumandeep Vidyapeeth. All participants provided written informed consent prior to enrollment. The cross-over design allowed each patient to serve as their own control, minimizing inter-individual variability and confounding factors.

## Sample size calculation and participant selection:

Sample size calculation was performed in consultation with a biostatistician using paired sample test parameters. Based on an anticipated mean difference of 1.0 mg/dL in pre-albumin levels between interventions, with standard deviation of 0.80, significance level (α) of 0.05 and desired power of 80%, the required sample size was determined to be five patients. Five completely edentulous patients (three males, two females; mean age 60 years) were recruited from individuals seeking prosthodontic treatment.

## Inclusion criteria comprised:

(1) complete edentulism of maxillary and mandibular arches for minimum six months; (2) residence within Vadodara region to ensure follow-up compliance; (3) minimum residual ridge dimensions of 10 mm height and 5 mm width in anterior mandible; (4) Class I maxillomandibular relationship; and (5) vegetarian dietary habits to minimize nutritional variability.

## Exclusion criteria included:

(1) systemic conditions contraindicating surgery; (2) psychiatric disorders or cognitive impairment; (3) pathology at proposed implant site including previous radiation, neoplasia, or bone disease; (4) tobacco use or smoking history; and (5) unwillingness to provide informed consent.

## Treatment protocol:

## Phase I: Baseline nutritional assessment:

Following initial screening, pre-treatment venous blood samples (10 mL) were collected under aseptic conditions from the median cubital vein for baseline nutritional marker analysis.

## Phase II: Conventional complete denture fabrication:

Maxillary and mandibular complete dentures were fabricated following established protocols. Primary impressions utilized medium-fusing impression compound in stock metal trays. Custom acrylic resin trays were fabricated with adequate spacing. Border molding employed low-fusing compound, with final impressions made using zinc oxide eugenol paste. Master casts were poured in Type III dental stone. Heat-cured acrylic resin record bases were constructed for jaw relation records. Maxillary casts were mounted on semi-adjustable articulators using facebow transfer. Centric relation records were verified using extraoral Gothic arch tracing. Balanced occlusion was established with semi-anatomic acrylic resin posterior teeth. Following try-in procedures confirming aesthetics, jaw relations and occlusion, dentures were processed using long-cycle heat-polymerization protocol. Finished dentures were inserted with appropriate adjustments and patients were instructed in denture hygiene and handling. After one-month adaptation period, blood samples were collected for nutritional reassessment.

## Phase III: Implant placement and overdenture conversion:

Pre-surgical evaluation included panoramic radiography and cone-beam computed tomography (DENTA scan) with radiopaque markers in the mandibular denture to assess bone quality, quantity and anatomical structures. Prophylactic antibiotic coverage (amoxicillin 2g or clindamycin 600mg) was administered one hour pre-operatively. Under local anaesthesia (2% lignocaine with 1:80,000 adrenaline), a crestal incision was made at the mandibular symphysis with buccal and lingual flap elevation. Sequential osteotomy preparation was performed using graduated twist drills under copious saline irrigation at 1620 rpm, following manufacturer protocols. Sand-blasted, large-grit, acid-etched (SLA) titanium implants (Kisses system, Biogenesis) ranging from 3.5-4.5 mm diameter and 11-13 mm length were placed flush with crestal bone. Healing abutments were connected, flaps were sutured with black braided silk and dentures were relieved and relined with tissue conditioner. Following three-month osseointegration period, ball attachments were connected to implants. Metal housings with O-ring retentive elements were picked up chairside using autopolymerizing acrylic resin. After one-month functional loading and dietary adaptation, final blood samples were collected.

## Nutritional marker analysis:

Venous blood samples were collected following standard phlebotomy protocols under tourniquet control with minimal stasis to prevent hemoconcentration. Samples were centrifuged and serum was separated for analysis. Pre-albumin was measured using turbidimetric immunoassay (SPINREACT reagent, Ark Diagnostics) on ERBA EM 200 analyzer, calibrated against reference materials (normal range: 20-40 mg/dL). Albumin was quantified using bromocresol green colorimetric method (ERBA reagent, Transasia Bio-medicals) on ERBA CHEM 7 analyzer (normal range: 3.5-5.2 g/dL). Vitamin B12 was analyzed via competitive enzyme immunoassay following automated pre-treatment (AIA-PACK B12, Tosoh Corporation) on AIA 360 system (normal range: 160-970 pg/mL). Serum iron was determined using chromazurol B photometric method (IRON Liquicolor, Human) on ERBA CHEM 5 Plus analyzer (normal range: Males 59-148 µg/dL, females 37-145 µg/dL). Haemoglobin was measured using non- cyanide method on SYSMEX KX-21 automated haematology analyzer with CELLPACK reagent (normal range: males 13.5-17.5 g/dL, females 12.0- 15.5 g/dL).

## Statistical analysis:

Data were analyzed using one-way analysis of variance (ANOVA) with repeated measures to compare nutritional markers across three time points: baseline (edentulous), post-CD (one month) and post-IOD (one month). Post-hoc pairwise comparisons were performed when overall F-test achieved significance. Confidence interval was set at 95% with significance threshold of p<0.05. All analyses were conducted using statistical software with consultation from a biostatistician.

## Results:

All five enrolled patients completed the study protocol without dropouts or adverse events. No implant failures, healing complications, or prosthetic complications occurred during the observation period. Mean values and standard deviations for all nutritional markers across the three intervention phases are presented in Table 1. Pre-albumin concentrations demonstrated progressive increase from baseline through both prosthetic interventions. One-way ANOVA revealed statistically significant differences among the three groups (F=20.84, p=0.0004). Pairwise comparisons showed significant improvements between baseline and conventional denture (mean difference 1.03 mg/dL, 95% CI: 0.67-1.40, p=0.001), baseline and implant overdenture (mean difference 2.40 mg/dL, 95% CI: 1.61-3.19, p=0.001) and conventional denture and implant overdenture (mean difference 1.37 mg/dL, 95% CI: 0.75-1.98, p=0.004). Albumin levels showed slight decrease from baseline to conventional denture, followed by recovery with implant overdenture. While overall ANOVA approached significance (F=3.89, p=0.052), post-hoc analysis revealed significant difference only between conventional denture and implant overdenture groups (mean difference 0.22 g/dL, 95% CI: 0.02-0.42, p=0.039). No significant differences were observed between baseline and conventional denture (p=0.271) or baseline and implant overdenture (p=0.876). Serum iron concentrations demonstrated progressive elevation across treatment phases. ANOVA indicated overall significant effect (F=4.92, p=0.031). Post-hoc testing revealed significant improvement from baseline to implant overdenture (mean difference 21.50 µg/dL, 95% CI: 7.12-35.88, p=0.014). The increase from baseline to conventional denture approached but did not achieve significance (mean difference 16.56 µg/dL, p=0.065), while the difference between conventional and implant overdentures was non-significant (p=0.270) (Table 2). Vitamin B12 concentrations showed minimal variation across treatment phases with slight non-significant decline (F=0.67, p=0.068). All values remained well within normal physiological ranges. Similarly, haemoglobin levels demonstrated no statistically significant changes among groups (F=0.52, p=0.612), with all measurements within gender-specific normal ranges throughout the study period. All implants achieved successful osseointegration without mobility or peri-implant complications. Patient compliance with denture hygiene protocols and follow-up appointments was excellent. No participants required implant removal or prosthesis remake during the observation period (Table 3).

## Discussion:

The proposed cross-over study will be the first study to determine the changes in nutritional status related to using central single implant-retained overdentures based on objective blood biochemical measurements. We observed considerable changes in sensitive nutritional parameters, especially pre-albumin and albumin, in response to single implant overdentures versus traditional complete dentures which proves the hypothesis that increased masticatory abilities are reflected in changes in objective nutritional benefits. The gradual rise in the pre-albumin levels during the treatment stages is a rather significant observation. A short half-life of about 48 hours, pre-albumin is a sensitive measure of the recent protein energy consumption and swift nutritional variations [16]. The large increase between baseline (19.82 mg/dl) and conventional denture (20.85 mg/dl) and further to implant overdenture (22.22 mg/dl) indicates that the quality and quantity of dietary protein were increasing with the improvement in masticatory efficiency. These findings are similar to the past studies which show that more effective chewing capacity allows one to consume high protein foods that are normally hard to chew with conventional dentures [17]. The weak pre-albumin level (around 20-40 mg/dL) of the baseline indicates the impaired nutritional condition that is quite common with edentulous elderly people. This observation supports epidemiological research recording poor dietary habits among dentures users because of limited food options and shunning of nutritious food that is hard to chew [6]. The improvements in pre-albumin levels to the normal range after the implant overdenture therapy indicate that the increased stability and retention of the prosthetic prosthesis directly promotes the improved intake of diet and the nutritional amount of the protein and energy. The pattern of albumin results was more complex, giving a paradoxical decrease of baseline to conventional denture and recovery with implant overdenture. Although this trend seems paradoxical at first, it is probably an indication of the longer half-life of albumin (around 20 days) and the time period of one month [15]. The high value between conventional and implant overdenture populations (p=0.039) suggests there is a relevant nutritional change of the enhanced prosthetic stability offered by such implant retention. The non-significance of the differences in baseline and implant overdenture groups (p=0.876) indicates that single implant overdentures help an individual with nutritional status to be restored to a pre-morbid state in well-nourished elderly people.

Past studies have indicated that there were great benefits on serum albumin in the elderly patients who wore two implant mandibular overdentures in comparison to the dentures used [9]. This evidence can be applied to single implant protocols, which might be the critical factor in which case, our findings indicate that it might not be the number of implants but anterior mandibular stabilization. This interpretation is justified by the biomechanical explanation, where a central placed implant in the symphyseal area offers resistance to anterior-posterior and rotational movements, the major direction of displacement of the dentures during mastication [13]. The outcome of the serum iron improvement between the baseline and the implant overdenture (p=0.014) has reflective clinical implications. Iron deficiency is a widespread nutritional issue among the elderly population, which leads to anaemia, tiredness and poor quality of life. The gradual rise in the level of iron (baseline 62.38 µg/dL, conventional denture 78.94 µg/dL, implant overdenture 83.88 µg/dL) indicates the consumption of iron-rich food improved. Although participants of the study were vegetarians, the bioavailability of iron through plant sources is very weak and therefore relies on how the food is prepared and the intake of the enhancing foods like vitamin C in fresh fruits and vegetables [15]. Improved masticatory performance was probably also an activity that allowed improved the consumption of the foods high in iron such as legumes, green leafy vegetables and whole grains that need effective chewing. The non-significant deviations in vitamin B12 and haemoglobin, though they all lie within the normal ranges are worthy of discussion. The metabolism of vitamin B12 is not straightforward and the level of this vitamin in the blood depends on the amount of gastric acid secreted, the production of intrinsic factor and the absorption of this vitamin at the end of the ileum as opposed to intake in the food [16]. The sources of vitamin B12 are mainly restricted to dairy products in the vegetarian population and foods that have been fortified, which do not demand much mastication. The slight non-significant decrease could be attributed to normal physiologic variability and not to the action of treatment. On the same note, the level of haemoglobin is determined by a combination of factors such as iron stores, vitamin B12, folate and erythropoietin production and the effects take longer periods before showing any changes as compared to the one month observation period that was adopted in this research [17]. The favorable experiences of the single implant overdentures in this study confirm past clinical reports that indicate high survival rates and satisfaction of patients with the single implant overdenture having minimalist design [11,12]. Mandibular symphysis offers the best anatomical sites in which the implants can be placed such as dense cortical bone, sufficient size and proximity to key neurovascular structures. The clinically successful protocol associated with immediate temporary denture relining after loading using tissue conditioner was without complications. The method has great benefits in regards to shorter treatment time and convenience to the patient and cost- effectiveness as opposed to delayed loading protocols that need extended healing time. Single implant overdentures are also a feasible treatment choice in the health economic context of the disadvantaged groups that cannot afford multiple implant process but would be willing to achieve better prosthetic stability and nutrition [18]. The high cost saving, relative to the two-implant overdentures, without loss of nutritional value as shown in this study, makes this an approach that is especially applicable to the scenario of public health applications and resource constraint environments. There are a number of shortcomings of this research that should be discussed. Can be considered a limitation, as it is necessary to use a small sample size (n=5) to statistically justify the primary outcome measure, but it cannot be generalized or offer any subgroup analysis. The one month observation period, it is quite enough to notice the changes in short half-life markers such as pre- albumin, but it might be insufficient enough to fully adapt to the diet and notice the alterations in long-term nutritional status markers. Although the cross-over design eliminates the inter-individual variability, it fails to provide the possible learning effects because patients became able to adjust their eating habits throughout the course of the research. Although the participants were kept on an exclusively vegetarian diet, which limits the number of potential confounding nutritional variations, this approach is not applicable to populations with mixed diets. Further studies are needed to include studies that are large multicenter studies with long-period follow-ups, a dietary intake studied through the use of validated food frequency questionnaires and quality of life to effectively determine the multidimensional advantages of single implant overdentures. A randomized controlled trial between single and two-implant protocols would give conclusive evidence on the best treatment processes. Research on economic viability and affordability in various socioeconomic groups would drive health policy and treatment recommendations among people.

## Conclusion:

We show that central single implant-retained mandibular overdentures have quantifiable nutritional advantages over traditional complete dentures. Marked positive changes in sensitive nutritional indicators especially pre-albumin and albumin depict increased protein-energy nutrition after the implant overdenture treatment. These objective biochemical data contribute to the clinical rationale of the idea of implant-assisted prosthetic rehabilitation as the way to enhance not only the oral activity and patient satisfaction but also the overall health and nutritional status in the whole body.

## Figures and Tables

**Table 1:** Descriptive statistics of nutritional markers across treatment phases

**Nutritional Marker**	**Baseline (Mean ± SD)**	**Conventional Denture (Mean ± SD)**	**Implant Overdenture (Mean ± SD)**	**Normal Range**
Pre-albumin (mg/dL)	19.82 ± 0.85	20.85 ± 0.95	22.22 ± 1.12	20-40
Albumin (g/dL)	4.03 ± 0.25	3.83 ± 0.22	4.05 ± 0.19	3.5-5.2
Vitamin B12 (pg/mL)	228.42 ± 24.04	221.90 ± 28.16	219.43 ± 19.82	160-970
Iron (µg/dL)	62.38 ± 18.35	78.94 ± 21.45	83.88 ± 24.20	37-148
Haemoglobin (g/dL)	13.02 ± 1.66	12.76 ± 1.58	13.14 ± 1.71	12.0-17.5

**Table 2:** ANOVA results and post-hoc pairwise comparisons for pre-albumin, albumin and iron

**Parameter**	**Comparison Groups**	**Mean Difference**	**95% CI**	**p-value**	**Significance**
Pre-albumin	Baseline vs CD	1.03	0.67 to 1.40	0.001	Yes
	Baseline vs IOD	2.4	1.61 to 3.19	0.001	Yes
	CD vs IOD	1.37	0.75 to 1.98	0.004	Yes
Albumin	Baseline vs CD	-0.2	-0.23 to 0.63	0.271	No
	Baseline vs IOD	0.02	-0.39 to 0.35	0.876	No
	CD vs IOD	0.22	0.02 to 0.42	0.039	Yes
Iron	Baseline vs CD	16.56	-1.66 to 34.78	0.065	No
	Baseline vs IOD	21.5	7.12 to 35.88	0.014	Yes
	CD vs IOD	4.94	-5.78 to 15.66	0.270	No

**Table 3:** ANOVA results for vitamin B12 and haemoglobin

**Parameter**	**Comparison Groups**	**Mean Difference**	**95% CI**	**p-value**	**Significance**
Vitamin B12	Baseline vs CD	-6.52	-35.86 to 22.82	0.571	No
	Baseline vs IOD	-8.99	-43.88 to 25.90	0.514	No
	CD vs IOD	-2.47	-12.56 to 7.62	0.534	No
Haemoglobin	Baseline vs CD	-0.26	-0.77 to 0.25	0.229	No
	Baseline vs IOD	0.12	-0.40 to 0.64	0.553	No
	CD vs IOD	0.38	-0.05 to 0.81	0.068	No
